# Construction of a cDNA library for miniature pig mandibular deciduous molars

**DOI:** 10.1186/1471-213X-14-16

**Published:** 2014-04-21

**Authors:** Tieli Song, Tingting Wu, Fulan Wei, Ang Li, Fu Wang, Yilin Xie, Dayong Liu, Zhipeng Fan, Xuejiu Wang, Shan Cheng, Chunmei Zhang, Junqi He, Songlin Wang

**Affiliations:** 1 Molecular Laboratory for Gene Therapy and Tooth Regeneration, Beijing Key Laboratory of Tooth Regeneration and Function Reconstruction, Capital Medical University School of Stomatology, Tian Tan Xi Li No.4, Beijing 100050, China; 2 Department of Stomatology, Beijing Tongren Hospital Affiliated to Capital Medical University, Dong Jiao Min Xiang No.1, Beijing 100730, China; 3 Department of Biochemistry and Molecular Biology, Capital Medical University School of Basic Medical Sciences, Beijing 100069, China

**Keywords:** Tooth, Development, Histology, Unigene, Sequence, Miniature pig

## Abstract

**Background:**

The miniature pig provides an excellent experimental model for tooth morphogenesis because its diphyodont and heterodont dentition resembles that of humans. However, little information is available on the process of tooth development or the exact molecular mechanisms controlling tooth development in miniature pigs or humans. Thus, the analysis of gene expression related to each stage of tooth development is very important.

**Results:**

In our study, after serial sections were made, the development of the crown of the miniature pigs’ mandibular deciduous molar could be divided into five main phases: dental lamina stage (E33-E35), bud stage (E35-E40), cap stage (E40-E50), early bell stage (E50-E60), and late bell stage (E60-E65). Total RNA was isolated from the tooth germ of miniature pig embryos at E35, E45, E50, and E60, and a cDNA library was constructed. Then, we identified cDNA sequences on a large scale screen for cDNA profiles in the developing mandibular deciduous molars (E35, E45, E50, and E60) of miniature pigs using Illumina Solexa deep sequencing. Microarray assay was used to detect the expression of genes. Lastly, through Unigene sequence analysis and cDNA expression pattern analysis at E45 and E60, we found that 12 up-regulated and 15 down-regulated genes during the four periods are highly conserved genes homologous with known Homo sapiens genes. Furthermore, there were 6 down-regulated and 2 up-regulated genes in the miniature pig that were highly homologous to Homo sapiens genes compared with those in the mouse.

**Conclusion:**

Our results not only identify the specific transcriptome and cDNA profile in developing mandibular deciduous molars of the miniature pig, but also provide useful information for investigating the molecular mechanism of tooth development in the miniature pig.

## Background

The pig is a large animal species suitable not only for meat production, but also as a model organism for comparative genomics and biomedical studies [1-6]. Due to the similarity of the dental and jaw bone system between human and pigs [7-9], using swine in dental biomedical research has increased in recent years, including research into dental implants, irradiation damage to parotid glands, bio-root regeneration, osteoradionecrosis, and bisphosphonate-related osteonecrosis, etc. [10-16].

The mouse is the most widely used animal model for studying tooth development. Almost all known molecular mechanisms of tooth formation and mineralization are derived indirectly or directly from studies of murine models [17-19]. However, mouse teeth are different from those of humans in both number and morphology, with only one dentition present throughout the mouse life cycle and a complete absence of canines and premolars [20]. Miniature pigs have both deciduous and permanent dentition, and all tooth types found in humans are present in pigs. However, detailed descriptive information concerning tooth development in the pig is lacking. Recently, our group has been dedicated to investigating the complicated mechanism of tooth development in miniature pigs, including the mRNA expression profiles of developing deciduous molar tooth [21], and the timing and sequencing of tooth replacement [22]. Other groups also reported that early morphogenesis of heterodont dentition can be divided into four significant stages in miniature pigs [23]. The purpose of the present study was to identify and classify the early stages of odontogenesis in miniature pig’s deciduous molar teeth, focusing on the differential expression of cDNAs during typical periods of tooth development. We also compared the genes from the E45 to E60 time course during tooth development with those of known Homo sapiens genes, and aimed to obtain basic information about their development for further molecular studies. We found that 12 up-regulated and 15 down-regulated genes may be involved in the miniature pig’s tooth development. We also found there were 6 down-regulated and 2 up-regulated genes with high homology to those in Homo sapiens, and compared these with those in mouse.

## Methods

### Ethics statement

Pregnant Wuzhishan miniature pigs were obtained from the Institute of Animal Science of the Chinese Agriculture University. Experiments were performed according to the Regulations for the Administration of Affairs Concerning Experimental Animals (Ministry of Science and Technology, China, revised in June 2004), and approved by the Animal Care and Use Committees of Capital Medical University, Beijing, China under permit No. CMU-B20100106. Animals were allowed access to food and water ad libitum under normal conditions and humanely sacrificed as necessary to ameliorate suffering. In brief, pregnant sows were anesthetized with a combination of 6 mg/kg ketamine chloride and 0.6 mg/kg xylazine, and pregnancy and the fetal state roughly determined by B-mode ultrasonography. After removing the fetuses by cesarean section, the pregnant sows were sacrificed by over-anesthetization.

### Preparation of tissues and histological staining

Developing miniature pig embryos were obtained by hysterectomy at embryonic days 30 (E30), E35, E40, E45, E50, E55, E60, and E65 according to the developmental progression of deciduous dentition in pigs [24]. After surgically removing the fetuses, germ tissue samples from deciduous molar teeth were removed from the mandibles under a microscope. The first mandibular molar could be obtained from E30. The second mandibular molar could be obtained from E35. The third mandibular molar could be obtained from E45. So the first deciduous mandibular molars were used in all studies. The samples were immediately frozen in liquid nitrogen and stored separately at -80°C until used for analysis. At least five miniature pig embryos were used for each evaluation. Specimens for the histological study were chosen by random selection from each specific age group litter. Embryo mandibles were separated and preserved in 4% paraformaldehyde. Mandible specimens from E30 were placed in EDTA bone decalcifying agent. Serial sections were made of the mandibular deciduous molar region. The tissues were mounted and stained with hematoxylin and eosin.

### RNA sample preparation and cDNA library establishment

Mandibular deciduous molar germs from E35, E45, E50, and E60 miniature pig embryos were excised and total RNA was extracted with an RNA purification kit (QIAGEN, Germany). RNA was then mixed in equal amounts from four different developmental time points. Oligo dT cellulose (MicroFast Track, Invitrogen, CA) was used as a template to synthesize first-strand cDNA. The cDNA library was constructed using the SMART cDNA Library Construction Kit (Clontech, CA). The obtained double-stranded (ds)-cDNA was purified using the QIAquick PCR Purification Kit (QIAGEN, Germany), then normalized with the DSN (duplex-specific nuclease) using the Trimmer-Direct Kit (Evrogen, Moscow, Russia). The normalized cDNAs were digested with Sfi I restriction enzyme, size fractionated (1–3 kb), directionally ligated into pDNR-LIB, and transformed into *E. coli* DH10B by electroporation. The cDNA library was plated on LB plates with X-gal, isopropyl-D-thiogalactopyranoside, and ampicillin. Thirty white colonies were randomly selected for identification of cDNA inserts in the recombinants to estimate the recombination efficiency. Exact same samples were used for both microarray and qRT-PCR.

### Microarray procedures

Microarray targets were prepared from each stage. RNA labelling, hybridization and scanning were conducted by a commercial Affymetrix array service (Institut de Recerca Hospital Universitari Vall d’Hebron, Barcelona, Spain). Reverse transcription of RNA and synthesis of biotin-labelled cRNA with one round of amplification were carried out following the standard Affymetrix one-cycle protocol according to the manufacturer's instructions. Samples were hybridized to the Affymetrix 24 K Genechip® Porcine Genome Array (Affymetrix, Santa Clara, CA, USA). Data analysis was performed with Bioconductor implemented in R 2.6.0 (http://cran.r-project.org/).

### Quantitative real-time RT-PCR

Total RNA reversely transcribed into cDNA using the PrimeScriptTM PT Reagent Kit (TaKaRa, Dalian, China). Amplifications of target genes were performed by real-time quantitative PCR (qPCR) using the cDNA as template, the specific primers and the SYBR® PrimeScript® RT–PCR Kit (Takara) on an ABI PRISM 7900 Real Time PCR System (Applied Biosystems, Carlsbad, USA). PCR amplifications were performed in duplicate at 95°C for 15 sec, and subjected to 40 cycles of 95°C for 5 sec 60°C for 30 sec, and 95°C for 15 sec 60°C for 15 sec 95°C for 15 sec. The primers used are shown in Additional file 1. The relative levels of target genes expression to the control of E45 were quantified. The relative levels of target gene mRNA transcripts to control β-actin were determined by 2^-ΔΔCt^.

### cDNA library sequencing, data processing, sequence analysis

After cDNA library identification, large-scale plasmid extraction and sequencing were performed for generation of expressed sequence tags (ESTs). High-quality ESTs were assembled into unigenes by phrap_0.990329 software. The unigene sequences were performed with E-values of less than 10^-5^ on the GenBank database according to the BLAST Search program (ftp://ftp.ncbi.nih.gov/blast/db/FASTA/). The unigenes were compared with annotations through the Gene Ontology Consortium using Interpro2GO. All ESTs were sequenced and analyzed at a commercial facility (BGI LifeTech Co. Ltd, Beijing, China). If the unigene sequence was more than 100 bp and its homology greater than 90% with a known functional pig gene, this gene was annotated in the pig genes. Then, if the sequence had high homology to a known gene in other species (E-values < 10^-5^), it was assumed that the gene is an orthologue of the comparator gene.

### Statistical analysis

Data of qRT-PCR are expressed as mean ± SEM. Data were analyzed by one-way analysis of variance. Multiple comparison between the groups was performed by using Bonferroni post-tests method. A p value of less than 0.05 was considered statistically significant. Statistical analysis was carried out using StatView 5.0 software (SAS Institute, Cary, NC) and GraphPad Prism 4.0 software.

## Results

### Development stages and histological characterization of miniature pig mandibular deciduous molars

**Figure 1:**
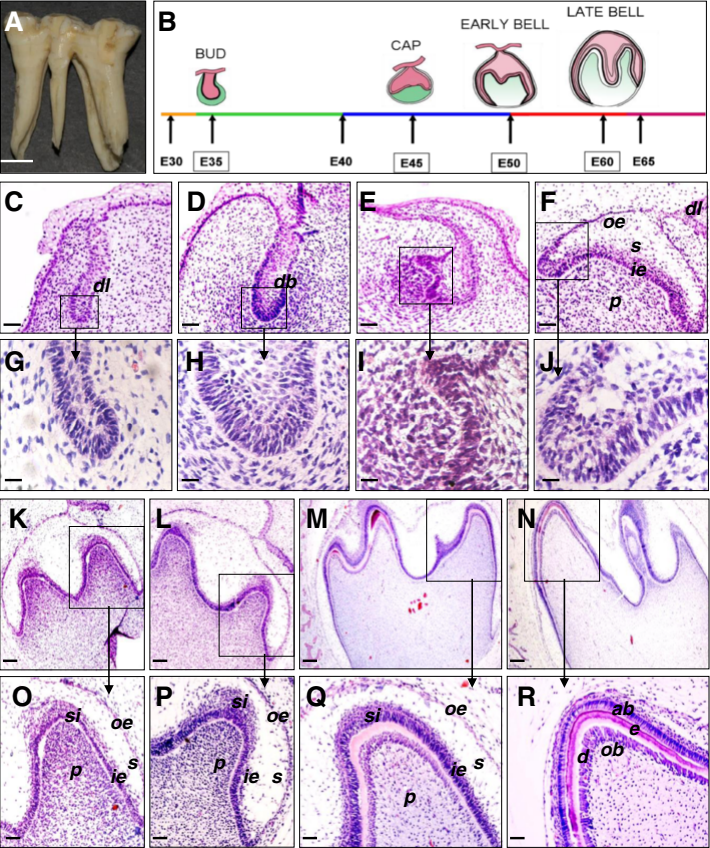
**Histology and stage of the developing miniature pig mandibular deciduous molar. (A)** Normal mandibular deciduous molar with 5 or 6 main cusps and 5 roots. **(B)** Developmental stages of the mandibular deciduous molar in the miniature pig. The relatively typical points in time are E35 (bud stage), E45 (cap stage), E50 (early bell stage), and E60 (late bell stage). **(C, G)** The epithelium grew into the mesenchyme to form the dental lamina (*dl*) at E30. **(D, H)** The dental lamina formed the dental bud (*db*) at E35. **(E, I)** The peripheral cells of the enamel organ extended outside at E40. (**F, J**) A typical cap stage appeared at E45, with differentiation of the outer enamel epithelial cells (*oe*), inner enamel epithelial cells (*ie*), and stellate reticular cells (*s*). The dental papilla (*p*) could also be observed. **(K, O)** At E50, the typical early bell stage was observed. The cusp morphology could be seen at the junction of the inner enamel epithelium and dental papilla. The stratum intermedium (*si*) appeared between the inner enamel epithelium and the stellate reticulum. **(L, P)** Morphological findings at E55. **(M, Q)** By E60, the molar reached late bell stage. In the cusp region, dental epithelial cells and mesenchymal cells were polarized and cells lengthened to become pre-ameloblast and pre-odontoblast. **(N, R)** Continuous and intact ameloblasts (*ab*), enamel (*e*), dentin (*d*), and odontoblasts (*ob*) were observed in the molar cusp at E65. Scale bars: 5 mm in A, 50 μm in C-F and O-R, 20 μm in G-J, and 100 μm in K-N.

Mandibular deciduous molar germs from E30 to E65 miniature pig embryos were excised (Additional file 2). Normal mandibular deciduous molars of miniature pigs have five or six main cusps and five roots (Figure 1A). Figure 1B shows the developmental stages of the mandibular deciduous molars. Development progressed as follows:

**E30:** In the E30 embryonic mandible, the oral epithelium thickened and extended to form the dental lamina (Figure 1C, G). **E35:** E35 samples showed hyperplasia of the lamina epithelium cells to form the primary enamel organ, meaning that the typical bud stage was observed (Figure 1D, H). The mesenchymal cells surrounding the bud clearly gathered. The placode was identified between the epithelium and the mesenchyme. **E40:** In the E40 mandibular region, the molar remained in the bud stage, but minor changes were seen at this time (Figure 1E, I). The peripheral cells of the enamel organ had now extended outside of the bud. **E45:** The typical cap stage for this molar did not appear until E45. At this time, the entire enamel looked like a cap (Figure 1F, J). More notable cell differentiation was present than at E40. Four cell types were identified; outer enamel epithelium, inner enamel epithelium, stellate reticulum, and dental papilla. The dental sac could also be observed. **E50:** E50 embryos showed the typical appearance of the early bell stage of this molar (Figure 1K, O). The dental papilla was larger than during the cap stage, whereas there were no morphological changes of dental papilla cells. The cusp morphology could be seen at the junction of the inner enamel epithelium and dental papilla. Inner enamel epithelial cells near the cusp region became stylolitic in shape, with the nucleolus far from the basalis. The stellate reticulum had sufficiently developed and the stratum intermedium appeared between the inner enamel epithelium and the stellate reticulum. **E55:** At this stage, there were no further changes except the adoption of a highly stylolitic shape by the inner enamel epithelial cells near the cusp region (Figure 1L, P). **E60:** By E60, the deciduous molar had reached the late bell stage of development (Figure 1M, Q). In the cusp region, dental epithelial cells and mesenchymal cells were polarized, and the cells lengthened to become pre-ameloblast and pre-odontoblast. At the same time, the pink matrix was seen in the cusp region. **E65:** At E65, continuous and intact ameloblasts, enamel, dentin, and odontoblasts were observed in the molar cusp (Figure 1N, R).

Taken together, the crown development of miniature pigs’ mandibular deciduous molar were divided into five main periods as follows (Figure 1B): the dental lamina stage (E33-E35), bud stage (E35-E40), cap stage (E40-E50), early bell stage (E50-E60), and late bell stage (E60-E65). The relatively typical time points are E35 (bud), E45 (cap), E50 (early bell), and E60 (late bell).

### Verification of gene expression

**Figure 2:**
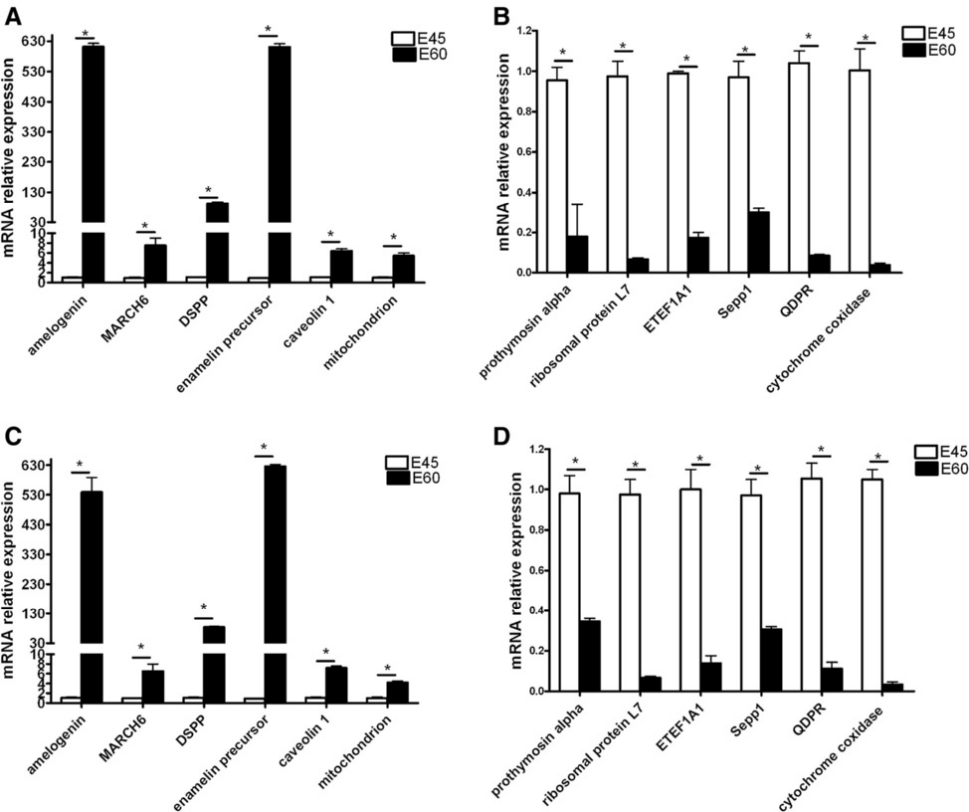
**mRNA expression of 12 genes detected by microarray and qRT-PCR.** The mRNA relative expression of 12 selected genes. **(A)** Amelogenin, membrane-associated ring finger 6 (MARCH6), isolate X269 mitochondrion, DSPP600 (DSPP), enamelin precursor, and caveolin were up-regulated at E60 compared with those at E45 in the microarray. **(B)** Ribosomal protein L7, eukaryotic translation elongation factor 1 alpha (EEF1A1 gene), prothymosin alpha, selenoprotein P (Sepp1), quinoid dihydropteridine reductase (QDPR) and cytochrome coxidase were down-regulated at E60 compared with those at E45 in the microarray. Validation of microarray data was achieved by using qRT-PCR. The six up-regulated and 6 down-regulated genes were changed accordingly (Figure 2C and 2D). **(C)** The six up-regulated genes were changed accordingly. **(D)** The six down-regulated genes were changed accordingly. Samples taken at E45 were used as controls. The relative levels of mRNA to GAPDH RNA were determined longitudinally by qRT-PCR assay. Data are expressed as mean relative values ± the standard error measurement (SEM) of each group of cells at each time point from three separate experiments. *P <0.05.

Amelogenin, membrane-associated ring finger 6 (MARCH6), isolate X269 mitochondrion, DSPP600 (DSPP), enamelin precursor, and caveolin were up-regulated at E45 compared with those at E60 in the microarray (Figure 2A). Ribosomal protein L7, eukaryotic translation elongation factor 1 alpha (EEF1A1 gene), prothymosin alpha, selenoprotein P (Sepp1), quinoid dihydropteridine reductase (QDPR) and cytochrome coxidase were down-regulated at E45 compared with those at E60 in the microarray (Figure 2B). Validation of microarray data was achieved by using qRT-PCR. The six up-regulated and down-regulated genes were changed accordingly (Figure 2C and 2D). The mRNA fold changes of all representative mRNAs were consistent with those in the normalized microarray data.

### cDNA library overview

Crown development in the miniature pig’s mandibular deciduous molars could be divided into four relatively typical periods as noted above. The mandibular deciduous molar germ cells were excised from miniature pig embryos at the E35, E45, E50, and E60 time points.

Total RNA from each period was used as template to synthesize cDNA and construct a cDNA library (Additional file 3 and Additional file 4). The titer of the unamplified cDNA library was approximately 3.0 × 10^5^ cfu/mL. A library comparison showed that all of the 30 selected clones had insert fragments, suggesting that the recombination rate was nearly 100% (Additional file 5). The primary cDNA library was used to generate ESTs. Twenty-three thousand and six hundred independent white clones were picked randomly for EST sequencing. A total of 20,065 ESTs were sequenced from the cDNA library. After removing the vector sequences and low-quality sequences (EST length less than 100 bp), 17,520 high-quality sequences were obtained with an average length of 441.61 bp, ranging from 100 to 681 nucleotides in length. Overall, 87.3% of the 17,520 high-quality sequences were longer than 300 bp. Cluster analyses assembled the 17,520 high-quality ESTs into 2,198 contigs and 11,709 singletons (13,907 unigenes, Table 1). The average length of the unigenes was 508 bp (range from 100 to 1,367 bp).

**Table 1:** A summary of ESTs and unigene analysis

**Description**	**Number**	**Percentage**
Total number of EST sequences	20,065	
Number of high quality sequences	17,520	87.3^1^
Number of singletons	11,709	58.3^1^
Number of contigs	2,198	10.9^1^
Number of unigenes	13,907	69.3^1^
Number of unigenes with BLAST hits	10,883	78.3^2^
Number of unknown unigenes	3,024	21.7^2^

Unigene Size: The Number of ESTs in Unigene, ^1^percentage of all ESTs, ^2^percentage of unigenes.

### Unigene sequence analysis

**Figure 3:**
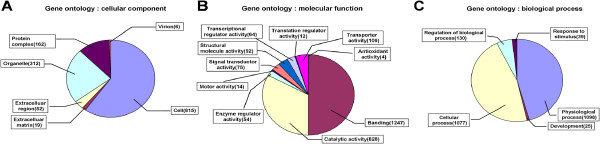
**Gene ontology (GO) graphs.** The unigene sequences were annotated using Interpro2GO software and included in the graphs. Each of the three categories is presented, including the cellular component **(A)**, molecular function **(B)**, and biological processes **(C)**.

Unigenes were compared to annotations through the Gene Ontology Consortium using Interpro2GO. Graphs based on the GO terms were created (Figure 3). Under the cellular component category, most transcripts were linked to inherent cellular structure, as well as to the protein complex (Figure 3A). In the category of molecular function, the five most abundant transcripts were involved in binding, catalytic activity, transporter activity, structural molecular activity, and signal transducer activity (Figure 3B). The most common biological processes were physiological processes and cellular processes (Figure 3C).

Based on BLAST results, 78.3% (10,883) of the unigenes were annotated to known genes, and 62.2% (6,772) had a BLAST score greater than 200. There were 3,024 unknown unigenes (21.7%) in the cDNA library. Unigenes whose sequences were markedly similar to known important proteins associated with dental development were found in this library, including ameloblastin, amelogenin, enamelin, dspp, and dmp1 (Additional file 6). What’s more, expression of known specific transcription factors (Additional file 7), growth factors (Additional file 8), and related receptors (Additional file 9) during murine tooth development also can be searched in the cDNA library. These results indicated that the cDNA library will be useful in facilitating further dental experiments in the miniature pig model.

Homology searches showed that the top ten species were as follows: Sus scrofa (5,771), Homo sapiens (2,122), Bos taurus (894), Equus caballus (467), Pan troglodytes (310), Canis familiaris (263), Macaca mulatta (262), Pongo abelii (53), Felis catus (39), and mouse (33). In the Unigene homology to Homo sapiens, 139 clones exhibited significant similarities to known genes (score greater than 500). Table 2 shows 23 unigenes with high homology to known Homo sapiens genes.

**Table 2:** Partial unigenes with high homology to Homo sapiens known genes

**Query name**	**Annotation**	**Score**
gdtca_Cluster6467	Homo sapiens cytoplasmic polyadenylation element binding protein 2 (CPEB2), transcript variant F, mRNA	1037
gdtca_Cluster1457	Homo sapiens zinc finger E-box binding homeobox 2 (ZEB2) on chromosome 2	944
gdtca_Cluster10676	Homo sapiens TGF-beta activated kinase 1/MAP3K7 binding protein 3 (TAB3), mRNA	914
gdtca_Cluster11351.seq.Contig1	Homo sapiens splicing factor, arginine/serine-rich 12 (SFRS12), transcript variant 2, mRNA	912
gdtca_Cluster8807	Homo sapiens zinc finger protein 407 (ZNF407) on chromosome 18	892
gdtca_Cluster2284	Homo sapiens SATB homebox 2 (SATB2) on chromosome 2	884
gdtca_Cluster4583	Homo sapiens SAPS domain family, member 3 (SAPS3), transcript variant 3, mRNA	846
gdtca_Cluster3803	Homo sapiens LUC7-like 3 (S. cerevisiae) (LUC7L3), transcript variant 1, mRNA	799
gdtca_Cluster4617	Homo sapiens fibronectin type III and SPRY domain containing 1-like (FSD1L), transcript variant 3, mRNA	797
gdtca_Cluster6088	Homo sapiens nebulette (NEBL), transcript variant 3, mRNA	783
gdtca_Cluster2858	Homo sapiens formin-like 3 (FMNL3), transcript variant 2, mRNA	773
gdtca_Cluster591	Homo sapiens fat mass and obesity associated (FTO) on chromosome 16	765
gdtca_Cluster11523.seq.Contig1	Homo sapiens Rho GTPase activating protein 19, mRNA (cDNA clone MGC:138804IMAGE:40082327) complete cds	763
gdtca_Cluster11577.seq.Contig1	Homo sapiens zinc finger CCCH-type containing 6 (ZC3H6), mRNA	729
gdtca_Cluster8595	Homo sapiens TEA domain family member 1 (SV40 transcriptional enhancer factor) (TEAD1), mRNA	729
gdtca_Cluster6631	Homo sapiens zinc finger and BTB domain containing 34 (ZBTB34), mRNA	706
gdtca_Cluster1697	Homo sapiens neuronal PAS domain protein 3 (NPAS3) on chromosome 14	686
gdtca_Cluster2466	Homo sapiens SIX homeobox 1 (SIX1) on chromosome 14	682
gdtca_Cluster1883	Homo sapiens B-cell CLL/lymphoma 11A (zinc finger protein) (BCL11A) on chromosome2	674
gdtca_Cluster8789	Homo sapiens TAR DNA binding protein (TARDPB) on chromosome 1	650
gdtca_Cluster4024	Homo sapiens protease, serine, 12 (neurotrupsin, motopsin) (PRSS12), mRNA	636
gdtca_Cluster10567	Homo sapiens fibroblast growth factor 14 (FGF14) on chromosome 13	620
gdtca_Cluster9160	Homo sapiens forkhead box D3 (FOX3D) on chromosome 1	618

### cDNA expression patterns during tooth development

**Figure 4:**
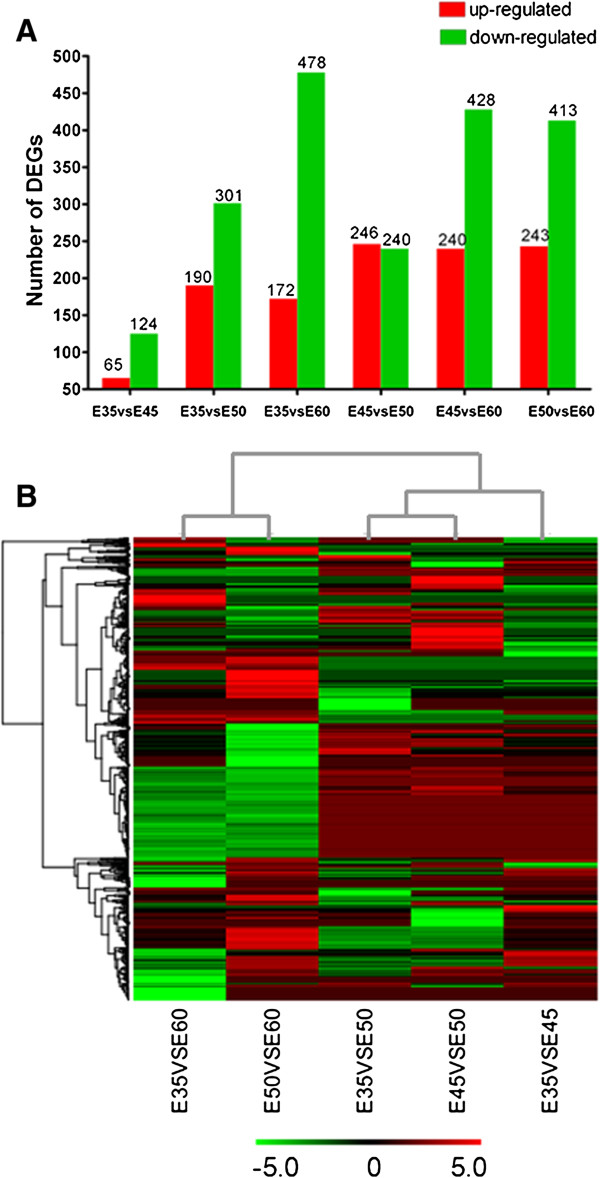
**Genes from the four time periods were compared with each other.** The differentially expressed genes from E35 vs E45, E35 vs E50, E35 vs E60, E45 vs E50, E45 vs E60, and E50 vs E60 were compared. **(A)** The number of up-regulated and down-regulated genes in each group is shown in the histogram. **(B)** The hierarchical clustering analysis of differentially expressed transcripts at different developmental stages.

We found that some cDNA sequences in the library had high homology to known Homo sapiens genes. Because detailed descriptive information concerning tooth development in Homo sapiens is lacking, miniature pigs are an optimal choice as a large animal model to investigate these molecular mechanisms. First, we compared all the four time periods with each other. The most differentially expressed genes were found between E35 and E60, followed by the genes between E45 and E60 (Figure 4A). And the fold change of all transcripts at E45 and E60 were the most significant (Figure 4B). Considering the germ tissue samples from E35 were small, and may have contained tissue from other nearby tissues, we blasted all the cDNA clones from the E45 and E60 time points against human genome DNA libraries. Twelve highly conserved genes were up-regulated (Table 3) and 15 highly conserved genes were down-regulated (Table 4). These results suggest that the 27 highly conserved genes may be involved in both miniature pig and Homo sapiens tooth development. Furthermore, 6 down-regulated (DPY30, ENAH, BORA, DAZAP2, NOP2, and DDX24) and 2 up-regulated genes (SHANK2, and CAMK2N1) in miniature pigs had higher homology to Homo sapiens genes compared with those in the mouse (Table 5).

**Table 3:** Up-regulated genes from E45 to E60 highly conserved homologous with known Homo sapiens genes

**Gene**	**TPM-E45**	**TPM-E60**	**log2 Ratio(E60/E45)**	**P-Value**	**FDR**	**Annotations**
gdtca_Cluster11155.seq.Contig1	8.33	51.11	2.61722	0	0	gi|119599067|schwannomin interacting protein 1, isoform CRA_c [Homo sapiens]
gdtca_Cluster8257	36.89	102.71	1.47727	0	0	gi|169161838|similar to hCG2040565 [Homo sapiens]
gdtca_Cluster3981	0.01	19.37	10.91961	2.22E-16	2.96E-15	gi|254911081|SH3 and multiple ankyrin repeat domains 2 (SHANK2), transcript variant 2, mRNAC1 [Homo sapiens]
gdtca_Cluster13121.seq.Contig1	49.06	101.57	1.04986	3.18E-13	3.26E-12	gi|119604964|hypothetical protein MGC2747, isoform CRA_c [Homo sapiens]
gdtca_Cluster11734.seq.Contig1	30.68	91.48	1.57616	5.29E-13	5.29E-12	gi|119588946|hCG1992991, isoform CRA_a [Homo sapiens]
gdtca_Cluster11879.seq.Contig1	12.96	28.65	1.14447	1.12E-10	9.21E-10	gi|119574191|hCG1983891 [Homo sapiens]
gdtca_Cluster7989	0.01	4.39	8.77808	3.49E-10	2.78E-09	gi|10834656|PP2281 [Homo sapiens]
gdtca_Cluster9754	3.97	13.35	1.74963	1.36E-09	1.01E-08	gi|7959776|PRO1489 [Homo sapiens]
gdtca_Cluster11696.seq.Contig1	11.77	24.9	1.08103	9.32E-09	6.53E-08	gi|221046286|unnamed protein product [Homo sapiens]
gdtca_Cluster6950	2.64	13.84	2.39023	2.22E-08	1.51E-07	gi|226528280|short coiled-coil protein isoform 1 [Homo sapiens]
gdtca_Cluster5165	0.01	1.95	7.60733	5.90E-05	0.00027	gi|187957136|LOC730130 protein [Homo sapiens]
gdtca_Cluster13166.seq.Contig1	1.85	5.7	1.62344	0.00018	0.0007	gi|6653742|7h3 protein [Homo sapiens]

**Table 4:** Down-regulated genes from E45 to E60 highly conserved homologous with known Homo sapiens genes

**Gene**	**TPM-E45**	**TPM-E60**	**log2 Ratio(E60/E45)**	**P-Value**	**FDR**	**Annotations**
gdtca_Cluster2986	2338.42	250.01	-3.22548	0	0	gi|37953286| transforming growth factor, beta 2 (TGFB2) [Homo sapiens]
gdtca_Cluster5775	6681.11	1182.18	-2.49864	0	0	gi|62897645|eukaryotic translation elongation factor 1 alpha 1 variant [Homo sapiens]
gdtca_Cluster6915	1033.21	228.2	-2.17876	0	0	gi|119625564|hCG1820575 [Homo sapiens]
gdtca_Cluster9994	176.92	24.09	-2.87659	6.37E-190	2.66E-188	gi|168984469|retinoblastoma binding protein 7 [Homo sapiens]
gdtca_Cluster6869	141.88	22.95	-2.62811	7.23E-139	2.68E-137	gi|14211889|protein dpy-30 homolog [Homo sapiens]
gdtca_Cluster12512.seq.Contig1	23.4	0.65	-5.16993	3.96E-40	8.93E-39	gi|119627667|poly(A) binding protein, cytoplasmic 4 (inducible form), isoform CRA_b [Homo sapiens]
gdtca_Cluster12827.seq.Contig1	41.12	10.42	-1.98048	6.12E-30	1.15E-28	gi|4507797|ubiquitin-conjugating enzyme E2v2 [Homo sapiens]
gdtca_Cluster12105.seq.Contig1	22.21	3.42	-2.69914	1.14E-23	1.87E-22	gi|242380880|hypothetical protein [Homo sapiens]
gdtca_Cluster12989.seq.Contig2	29.88	7.32	-2.02926	8.32E-23	1.34E-21	gi|34533983|unnamed protein product [Homo sapiens]
gdtca_Cluster12482.seq.Contig1	6.35	0.01	-9.31061	4.44E-13	4.48E-12	gi|166014265|enabled-like protein variant hMenaDv6 [Homo sapiens]
gdtca_Cluster5336	16.26	4.72	-1.78447	3.12E-11	2.64E-10	gi|158256424|unnamed protein product [Homo sapiens]
gdtca_Cluster5548	9.26	1.95	-2.24754	7.36E-09	5.19E-08	gi|4929627|CGI-79 protein [Homo sapiens]
gdtca_Cluster11214.seq.Contig1	4.63	0.49	-3.2401585391	9.20E-07	5.23E-06	gi|211904140|DAZ-associated protein 2 isoform c [Homo sapiens]
gdtca_Cluster8769	8.86	2.77	-1.67742	2.96E-06	1.59E-05	gi|119578911|nuclear transcription factor, X-box binding 1, isoform CRA_a [Homo sapiens]
gdtca_Cluster919	7.93	2.93	-1.43642	7.92E-05	0.00035	gi|119609186|nucleolar protein 1, 120kDa [Homo sapiens]

**Table 5:** Genes only expressed in Homo sapiens during the tooth development course of miniature pigs

	**Query**	**Gene**
Down-regulation	gdtca_Cluster6869	DPY30
	gdtca_Cluster12482.seq.Contig1	ENAH
	gdtca_Cluster5336	BORA
	gdtca_Cluster11214.seq.Contig1	DAZAP2
	gdtca_Cluster919	NOP2
	gdtca_Cluster9198	DDX24
Up-regulation	gdtca_Cluster3981	SHANK2
	gdtca_Cluster9754	CAMK2N1

## Discussion

In the present study, we constructed a cDNA library from miniature pig molar tissue over the period of tooth development. We then confirmed the fold change of gene expression using qRT-PCR. Using large-scale sequencing and ESTs assemblage, a large pool of unigenes were found in this library. A total of 13,907 unigenes were assembled from 17,520 ESTs, indicating that redundancy was only 20.6%. Furthermore, 95% of these Unigenes contain only one or two ESTs, indicating the positive effect of cDNA library normalization, which can be used to identify expressed genes in the future.

Great progress has been made in the study of molecular mechanisms during tooth morphogenesis in the past 20 years, and most data were derived from studies on rodent embryos [19]. However, owing to its similarity to human anatomy and physiology, pig models are superior in many aspects for the study of human development, diseases, and pre-clinical therapies [4-6]. Both domestic pigs and miniature pigs can be used in medical experimentation, but miniature pigs have many advantages, including an inherently small size, early sexual maturity, rapid breeding, and ease of management [25,26]. The deciduous molar in the Chinese experimental miniature pig is oblong in shape and has five or six main cusps. It is bigger and has different morphology compared with all other deciduous teeth in the mandible, and it lies on the end of mandible body. All these characteristics contribute to being able to easily and accurately distinguish and isolate the tooth germ. There are high correlations between the deciduous and permanent teeth [27]. Therefore, the deciduous molar was chosen as the first model tooth to evaluate in miniature pig tooth development.

There is little information concerning tooth development in large animal models [23,24]. Some sequences in this cDNA library had high similarity with proteins associated with dental development such as ameloblastin, amelogenin, enamelin, dspp, and dmp1 [28-31]. Many genes involved in tooth development remain to be identified. For example, unigenes with high homology to known Homo sapiens genes in this library included *FOXD3, SATB2, ZEB2* (Zinc finger E-box-binding homeobox 2 gene), etc. FOXD3, a member of the forkhead family of transcriptional regulations, plays a role in maintaining the epiblast and its derivatives and in establishing pluripotent ESC lines [32]. SATB2 is a recently cloned member of the family of special AT-rich binding proteins. Satb2^-/-m^ice exhibit both craniofacial abnormalities that resemble those observed in humans carrying a SATB2 translocation and defects in osteoblast differentiation and function [33]. ZEB2 has been involved in Mowat-Wilson syndrome (MWS), a multiple congenital anomaly syndrome characterized by a distinct facial phenotype. MWS is caused by heterozygous mutations or deletions in ZEB2 [34].

Data from this study will facilitate further dental experiments in the miniature pig model. In the present study, we found that 12 up-regulated and 15 down-regulated genes may be involved in the miniature pig’s tooth development. We also found 6 down-regulated (DPY30, ENAH, BORA, DAZAP2, NOP2, and DDX24) and 2 up-regulated genes (SHANK2 and CAMK2N1) in miniature pigs with higher homology to Homo sapiens genes compared with those in the mouse. SHANK2 is a member of the Shank family of synaptic proteins that function as molecular scaffolds in the postsynaptic density [35]. CAMK2N1 (calcium/calmodulin-dependent protein kinase II) expresses at high levels in osteogenic cells, and may be a good marker of osteogenic differentiation in mesenchymal stem cells [36]. There is very little known about these genes and their roles in tooth development. Investigating the functions of these genes in tooth development in a swine model and humans will be of great interest.

In summary, we evaluated the histological features of miniature pigs’ deciduous molar development and identified five primary phases. A miniature pig embryo tooth cDNA library was constructed, which contains approximately 3.0 × 10^5^ cfu with 17,520 high quality EST sequences and 13,907 unigenes. The established cDNA library provides the basis for further tooth development studies using this animal model.

## Conclusion

Our results not only identify the specific transcriptome and cDNA profile in developing mandibular deciduous molars of the miniature pig, but also provide useful information for investigating the molecular mechanism of tooth development in the miniature pig.

### Availability of supporting data

The supporting data is available in the Genebank. The library accession numbers is LIBEST_028375. And the Library name is developing mandibular deciduous molars of the miniature pig cDNA library.

## Competing interests

The authors declare that they have no competing interests.

## Authors’ contribution

TS, TW, FW, and AL participated in initial discovery and design. TS, FW, and YX performed the histology experiments and carried out the gene expression assays. TS, TW and SW analyzed the data and wrote the manuscript. ZF, DL, XW, SC, CZ, and JH participated in the collection of the data and performed the statistical analysis. SW conceived of the study, participated in its design and coordination and got funding. All authors read and approved the final manuscript.

## Supplementary Material

Additional file 1 Primers used for qRT-PCR. Click here for file

Additional file 2 **Excised mandibular deciduous molar germs from E35 and to E45 in miniature pig embryos. (A, B)** The miniature pig mandible at E35. Red circles indicate the mandibular deciduous molar area. **(C)** One side of the mandible of a E45 miniature pig embryo. **(D)** Stripped medial mandible. **(E)** Mandibular stripped of excess tissue. **(F)** Isolated mandibular deciduous molar germs. Green arrow indicates mandible; red arrow indicates germ. Click here for file

Additional file 3 **Agarose gel electrophoresis of total RNA.** Total RNA was extracted from the mandibular deciduous molar germs at each developmental stage (E35, E45, E50, E60). Total RNA examined by electrophoresis on 1.1% agarose gels showed two bright bands at 28S rRNA and 18S rRNA; the former was equal to or more abundant than the latter, indicating that little or no RNA degradation or contamination occurred during isolation. Click here for file

Additional file 4 **Agarose gel electrophoresis of double-stranded cDNA after PCR.** One μg (1 μl) of poly(A)^+^ RNA was used as RNA template in first-strand synthesis. A volume of 2 μl of single-stranded cDNA served as a template for primer-extension-based, second-stand synthesis using 21 thermal cycles. Lane M: DL2000 plus marker (Transgen, 5 μl). Lane 1: 5 μl sample of the double-stranded cDNA product showing a smear ranging from 0.1 to 3 kb. Click here for file

Additional file 5 **Agarose gel electrophoresis of the PCR products from randomly selected cDNA inserts (30 plaques) from the unamplified cDNA library.** The size of PCR products were between 1 ~ 3 kb for 30 samples. Lane M: DL2000 plus marker (Transgen). Click here for file

Additional file 6 Known specific protein matrix expression in mice searched in the cDNA library during tooth development. Click here for file

Additional file 7 Known specific transcription factor expression in mice searched in the cDNA library during tooth development. Click here for file

Additional file 8 Known growth factor expression in mice searched in the cDNA library during tooth development. Click here for file

Additional file 9 Known related receptor expression in mice searched in the cDNA library during tooth development. Click here for file
