# Supplementary material for: Construction of a cDNA library for miniature pig mandibular deciduous molars
Source: BMC Dev Biol. 2014 Apr 21;14:16. doi: 10.1186/1471-213X-14-16 (PMC4021421; doi:10.1186/1471-213X-14-16)
Supplement: Additional file 1 — Primers used for qRT-PCR. [file 1471-213X-14-16-S1.doc]

| gene  Additional file 1 The primers of 12 selected genes | Forward (5’-3’) | Backward (5’-3’) |
| --- | --- | --- |
| gdtca_Cluster5497 | GCCATTTCTTTTGCCTTCAG | CCGCCACTTATTTTCCACTG |
| gdtca_Cluster10357 | TGGTTACGGCAAAATCAACA | CAGCATCTCCCCCTTCTACA |
| gdtca_Cluster5775 | AATGGTGACAACATGCTGGA | ATGCCAGGTTTGAGAACACC |
| gdtca_Cluster6973 | AGGCAGGAAGGTGAAAATGA | TTGCACAAATCCAATTCCAA |
| gdtca_Cluster5390 | ACGCCCTTAGAATTCCTGGT | CCCACGGATGGATTAATGAC |
| gdtca_Cluster11276.seq.Contig1 | AGGCAGGAAGGTGAAAATGA | TTGCACAAATCCAATTCCAA |
| gdtca_Cluster993 | TGAGGAGATGATGCGCTATG | TTTGCTCTGCTGTTTGGATG |
| gdtca_Cluster7237 | AACGAGGATGTTGGAAATGC | TTATCCAGGCCAGCAAATTC |
| gdtca_Cluster10997.seq.Contig1 | GCAGTCTCTGCTTCCTGACC | TGGTAGGCACAAATCATCCA |
| gdtca_Cluster3581 | GAGTTGTCCCGCTTCTTCTG | GATGCCATTTGCGTAAACCT |
| gdtca_Cluster1026 | CGAGAAAGCACTTTCCAAGG | CTAATTCGGGTGTTGGTGCT |
| gdtca_Cluster11553.seq.Contig1 | TAGGTCAGCAGCCTCCCTAA | CTGGTGAGAGGCAGGAAAAG |
