# Supplementary figures and images for: Construction of a cDNA library for miniature pig mandibular deciduous molars
Source: BMC Dev Biol. 2014 Apr 21;14:16. doi: 10.1186/1471-213X-14-16 (PMC4021421; doi:10.1186/1471-213X-14-16)

## Slide 1
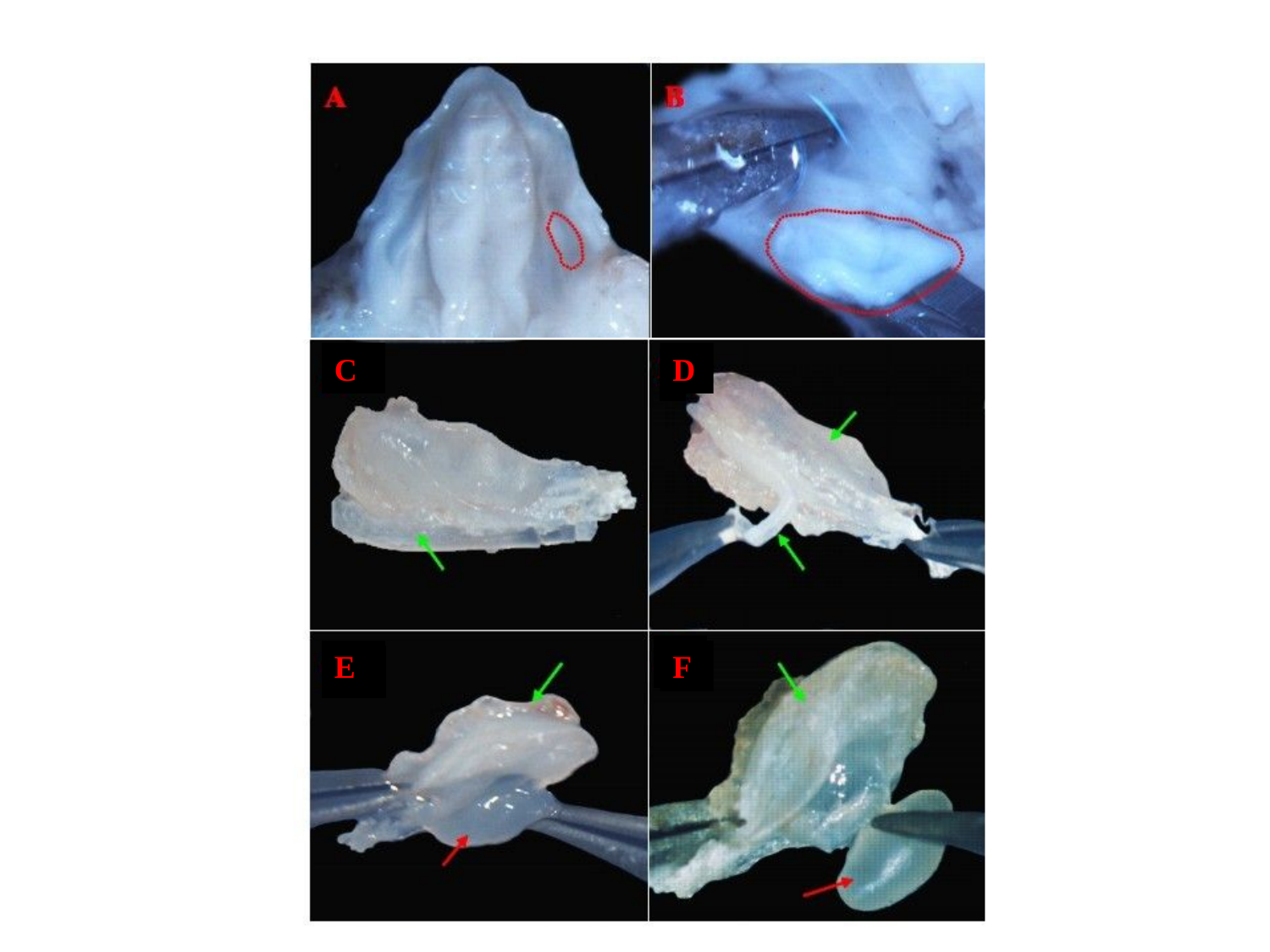

C
D
E
F

Supplement: Additional file 2 — Excised mandibular deciduous molar germs from E35 and to E45 in miniature pig embryos. (A, B) The miniature pig mandible at E35. Red circles indicate the mandibular deciduous molar area. (C) One side of the mandible of a E45 miniature pig embryo. (D) Stripped medial mandible. (E) Mandibular stripped of excess tissue. (F) Isolated mandibular deciduous molar germs. Green arrow indicates mandible; red arrow indicates germ. [file 1471-213X-14-16-S2.pptx]

## Slide 1
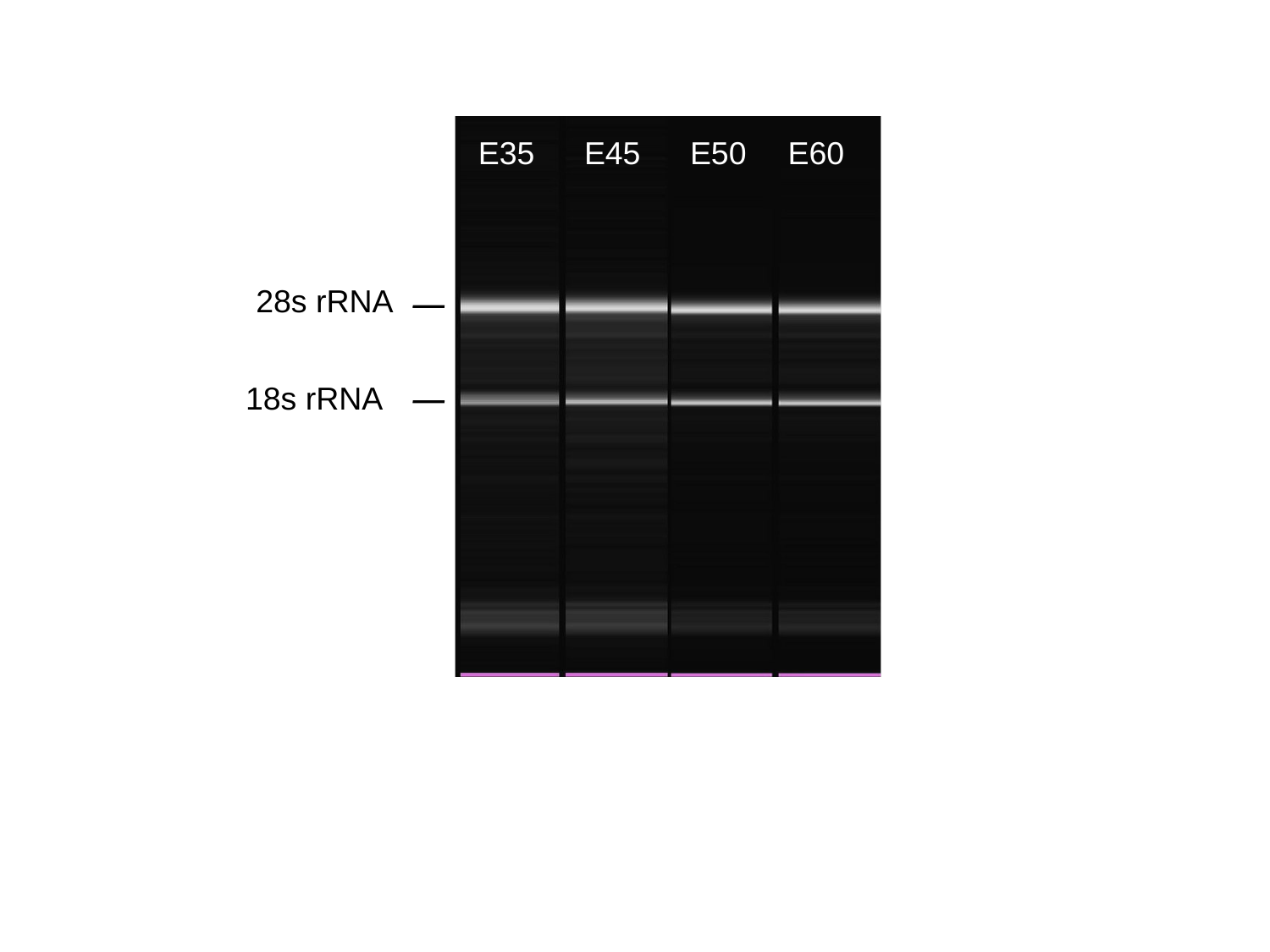

E35
E45
E50
E60
28s rRNA
18s rRNA

Supplement: Additional file 3 — Agarose gel electrophoresis of total RNA. Total RNA was extracted from the mandibular deciduous molar germs at each developmental stage (E35, E45, E50, E60). Total RNA examined by electrophoresis on 1.1% agarose gels showed two bright bands at 28S rRNA and 18S rRNA; the former was equal to or more abundant than the latter, indicating that little or no RNA degradation or contamination occurred during isolation. [file 1471-213X-14-16-S3.pptx]

## Slide 1
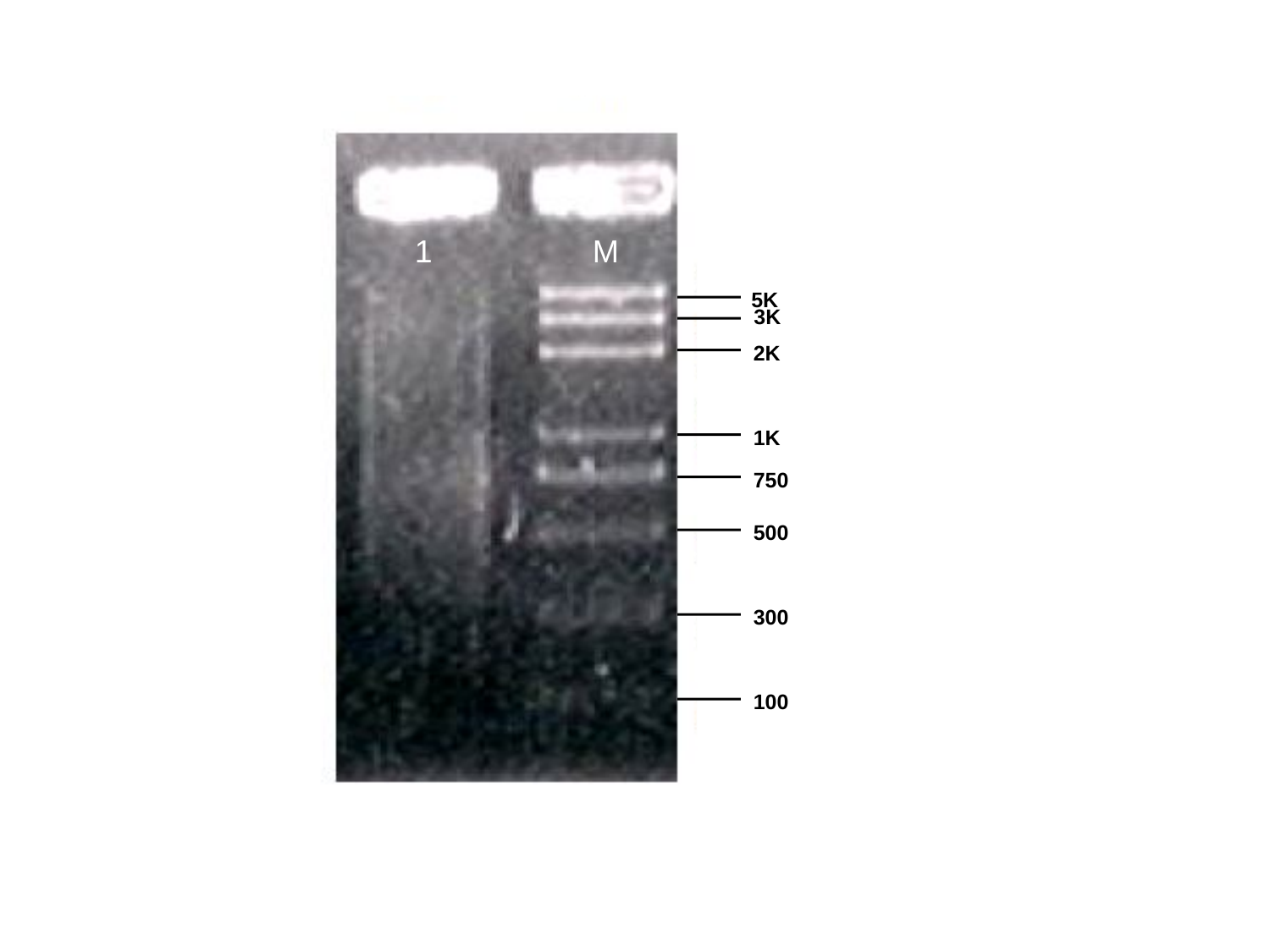

1
M
5K
3K
2K
1K
750
500
300
100

Supplement: Additional file 4 — Agarose gel electrophoresis of double-stranded cDNA after PCR. One μg (1 μl) of poly(A)+ RNA was used as RNA template in first-strand synthesis. A volume of 2 μl of single-stranded cDNA served as a template for primer-extension-based, second-stand synthesis using 21 thermal cycles. Lane M: DL2000 plus marker (Transgen, 5 μl). Lane 1: 5 μl sample of the double-stranded cDNA product showing a smear ranging from 0.1 to 3 kb. [file 1471-213X-14-16-S4.pptx]

## Slide 1
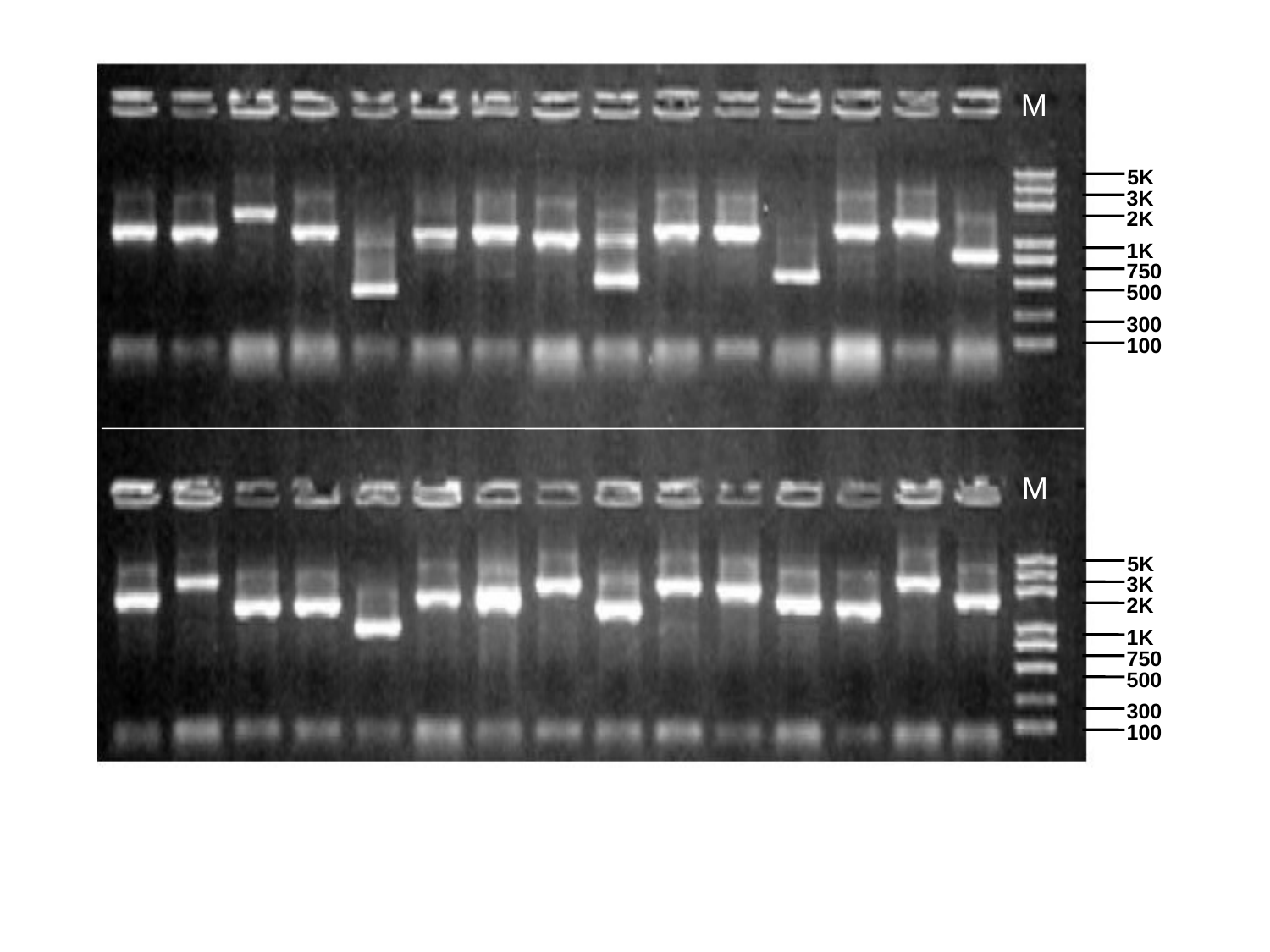

M
5K
3K
2K
1K
750
500
300
100
M
5K
3K
2K
1K
750
500
300
100

Supplement: Additional file 5 — Agarose gel electrophoresis of the PCR products from randomly selected cDNA inserts (30 plaques) from the unamplified cDNA library. The size of PCR products were between 1 ~ 3 kb for 30 samples. Lane M: DL2000 plus marker (Transgen). [file 1471-213X-14-16-S5.pptx]
