# Supplementary material for: Construction of a cDNA library for miniature pig mandibular deciduous molars
Source: BMC Dev Biol. 2014 Apr 21;14:16. doi: 10.1186/1471-213X-14-16 (PMC4021421; doi:10.1186/1471-213X-14-16)
Supplement: Additional file 6 — Known specific protein matrix expression in mice searched in the cDNA library during tooth development. [file 1471-213X-14-16-S6.doc]

Additional file 6. Known specific protein matrix expression in mice searched in the cDNA library during tooth development

|  | M | P | annotation | id | unigene |
| --- | --- | --- | --- | --- | --- |
| ameloblastin | ＋ | ＋ | Sus scrofa ameloblastin (AMBN), mRNA gi|47522893|ref|NM_214037.1|） | 99 | gdtca_Cluster13268.seq.Contig3 |
| amelogenin | ＋ | ＋ | Sus scrofa amelogenin 173A mRNA, （gi|47522675|ref|NM_213906.1|） | 99 | gdtca_Cluster10997.seq.Contig1 |
| enamelin | ＋ | ＋ | Sus scrofa enamelin (ENAM) gene, （gi|255928772|gb|GQ354856.1|） | 100 | gdtca_Cluster11289.seq.Contig1 |
| DSPP | ＋ | ＋ | Sus scrofa DSPP600 (DSPP) mRNA, （gi|47523289|ref|NM_213777.1|） | 99 | gdtca_Cluster7237 |
| DMP1 | ＋ | ＋ | Sus scrofa dentin (DMP1), mRNA（gi|194018671|ref|NM_001129953.1|） | 99 | gdtca_Cluster11004.seq.Contig1 |

M（mouse） P（pig） id （identity）
