# Supplementary material for: Construction of a cDNA library for miniature pig mandibular deciduous molars
Source: BMC Dev Biol. 2014 Apr 21;14:16. doi: 10.1186/1471-213X-14-16 (PMC4021421; doi:10.1186/1471-213X-14-16)
Supplement: Additional file 7 — Known specific transcription factor expression in mice searched in the cDNA library during tooth development. [file 1471-213X-14-16-S7.doc]

| Additional file 7. Known specific transcription factor expression in mice searched in the cDNA library during tooth development | M | P | annotation | id | unigene |
| --- | --- | --- | --- | --- | --- |
| [Ahr](http://bite-it.helsinki.fi/AHRR.htm) | ＋ | ＋ | Sus scrofa aryl hydrocarbon receptor nuclear translocator-like (ARNTL), mRNA （gi|147898768|ref|NM_001097425.1|） | 98 | gdtca_Cluster11260.seq.Contig1 |
| [Arnt](http://bite-it.helsinki.fi/ARNT.htm) | ＋ | ＋ | Sus scrofa aryl hydrocarbon receptor nuclear translocator,transcript variant2(ARNT),mRNA （gi|194036274|ref|XM_001929670.1|） | 98 | gdtca_Cluster12146.seq.Contig1 |
| [Barx1](http://bite-it.helsinki.fi/BARX1.htm) | ＋ | － |  |  |  |
| [C-Myb](http://bite-it.helsinki.fi/C-MYB.htm) | ＋ | ＋ | Bos taurus myosin binding protein C, mRNA complete cds (gi|160425242|ref|NM_001110773.1|) | 91 | gdtca_Cluster12829.seq.Contig1 |
| [catenin beta](http://bite-it.helsinki.fi/BCATENIN.htm) | ＋ | ＋ | Sus scrofa catenin mRNA for beta-catenin,complete cds(gi|47523791|ref|NM_214367.1) | 94 | gdtca_Cluster13269.seq.Contig2 |
| [Dermo1](http://bite-it.helsinki.fi/DERMO1.htm) | ＋ | ＋ | Homo sapiens twisted gastrulation homolog 1 (Drosophila) (TWSG1), mRNA (gi|254750702|ref|NM_020648.5|) | 92 | gdtca_Cluster6019 |
| [Dlx1](http://bite-it.helsinki.fi/DLX1.htm) | ＋ | ＋ | Bos taurus distal-less homeobox 1 （DLX1), mRNA(gi|149642882|ref|NM_001099017.1|) | 83 | gdtca_Cluster13109.seq.Contig1 |
| [Dlx2](http://bite-it.helsinki.fi/DLX2R.htm) | ＋ | ＋ | Homo sapiens distal-less homeobox 2 (DLX2) on chromosome 2(gi|219521819|ref|NG_009219.1|) | 94 | gdtca_Cluster7003 |
| [Dlx3](http://bite-it.helsinki.fi/DLX3.htm) | ＋ | － |  |  |  |
| [Dlx4](http://bite-it.helsinki.fi/DLX7R.htm) | ＋ | － |  |  |  |
| [Dlx5](http://bite-it.helsinki.fi/DLX5R.htm) | ＋ | ＋ | Homo sapiens distal-less homeobox 5 (DLX5) on chromosome 7(gi|219521820|ref|NG_009220.1|) | 88 | gdtca_Cluster12958.seq.Contig1 |
| [Dlx6](http://bite-it.helsinki.fi/DLX6R.htm) | ＋ | ＋ | Bos taurus similar to distal-less homeobox 6 (DLX6), mRNA(gi|194666189|ref|XM_001254126.2|) | 90 | gdtca_Cluster245 |
| [Egr1](http://bite-it.helsinki.fi/EGR1.htm) | ＋ | － | Human DNA sequence Contains part of the EGR gene for early growth response 2 (Krox-20 homolog, Drosophila), complete sequence(gi|16972892|emb|AL357125.22|) | 90 | gdtca_Cluster7914 |
| [forkhead](http://bite-it.helsinki.fi/FKH6.htm) | ＋ | ＋ | Homo sapiens forkhead box D3 (FOXD3) on chromosome 1(gi|238018120|ref|NG_012220.1|) | 98 | gdtca_Cluster9160 |
| [Gli1](http://bite-it.helsinki.fi/GLI1.htm) | ＋ | － |  |  |  |
| [Gli2](http://bite-it.helsinki.fi/GLI2.htm) | ＋ | － |  |  |  |
| [Gli3](http://bite-it.helsinki.fi/GLI3.htm) | ＋ | ＋ | Homo sapiens GLI family zinc finger 3 (GLI3) on chromosome 7(gi|197333864|ref|NG_008434.1|) | 100 | gdtca_Cluster802 |
| [Hand1](http://bite-it.helsinki.fi/HAND1.htm) | ＋ | － |  |  |  |
| [Hand2](http://bite-it.helsinki.fi/HAND2M.htm) | ＋ | － |  |  |  |
| [Hes1](http://bite-it.helsinki.fi/HES1.htm) | ＋ | － |  |  |  |
| [Hes5](http://bite-it.helsinki.fi/HES5.htm) | ＋ | － |  |  |  |
| [Irf6](http://bite-it.helsinki.fi/IRFMW.htm) | ＋ | ＋ | Sus scrofa interferon regulatory factor 6 (IRF6), mRNA(gi|47523621|ref|NM_214278.1|) | 98 | gdtca_Cluster5558 |
| [Irx1](http://bite-it.helsinki.fi/IRX1R.htm) | ＋ | － |  |  |  |
| [Irx2](http://bite-it.helsinki.fi/IRX2R.htm) | ＋ | － |  |  |  |
| [Irx3](http://bite-it.helsinki.fi/IRX3R.htm) | ＋ | － |  |  |  |
| [Irx4](http://bite-it.helsinki.fi/IRX4R.htm) | ＋ | － |  |  |  |
| [Irx5](http://bite-it.helsinki.fi/IRX5R.htm) | ＋ | － |  |  |  |
| [Irx6](http://bite-it.helsinki.fi/IRX6R.htm) | ＋ | － |  |  |  |
| [Islet1](http://bite-it.helsinki.fi/ISLE1I.htm) | ＋ | － |  |  |  |
| [Lef1](http://bite-it.helsinki.fi/LEF1.htm) | ＋ | ＋ | Sus scrofa lymphoid enhancer-binding factor 1 (LEF1), mRNA( gi|194018691|ref|NM_001129967.1|) | 99 | gdtca_Cluster11234.seq.Contig1 |
| [Lhx6](http://bite-it.helsinki.fi/LHX6.htm) | ＋ | ＋ | Homo sapiens LIM homeobox 6 (LHX6), transcript variant 2, mRNA(gi|134284360|ref|NM_199160.2|) | 87 | gdtca_Cluster5655 |
| [Lhx8](http://bite-it.helsinki.fi/LHX7.htm) | ＋ | ＋ | Sus scrofa LIM homeobox 8 (Lhx8) mRNA, complete cds(gi|261490775|ref|NM_001166315.1|) | 99 | gdtca_Cluster10010 |
| [Msx1](http://bite-it.helsinki.fi/DMMSX1.htm) | ＋ | ＋ |  |  |  |
| [Msx2](http://bite-it.helsinki.fi/MSX2.htm) | ＋ | ＋ |  |  |  |
| [N-myc](http://bite-it.helsinki.fi/N-MYC.htm) | ＋ | ＋ |  |  |  |
| [Oasis](http://bite-it.helsinki.fi/OASIS.htm) | ＋ | ＋ | Equus caballus cAMP responsive element binding protein 5, transcript variant 1 (CREB5), mRNA(gi|194209758|ref|XM_001500010.2|) | 92 | gdtca_Cluster12646.seq.Contig1 |
| [Osr2](http://bite-it.helsinki.fi/OSR2R.htm) | ＋ | － |  |  |  |
| [Pax9](http://bite-it.helsinki.fi/DMPAX9.htm) | ＋ | ＋ |  |  |  |
| [pigpen](http://bite-it.helsinki.fi/PIGPEN.htm) | ＋ | ＋ | Homo sapiens fusion (involved in t(12;16) in malignant liposarcoma) (FUS) on chromosome 16(gi|258547117|ref|NG_012889.1|) | 95 | gdtca_Cluster11829.seq.Contig1 |
| [Pitx2](http://bite-it.helsinki.fi/OTLX2.htm) | ＋ | － |  |  |  |
| [Plu1](http://bite-it.helsinki.fi/PLU1R.htm) | ＋ | ＋ | Bos taurus similar to retinoblastoma-binding protein 6, transcript variant 2 (RBBP6), mRNA(gi|194678600|ref|XM_001254138.2|) | 92 | gdtca_Cluster12539.seq.Contig1 |
| [Prrx1](http://bite-it.helsinki.fi/PRX1.htm) | ＋ | － |  |  |  |
| [Prrx2](http://bite-it.helsinki.fi/PRX2P.htm) | ＋ | － |  |  |  |
| [Runx1](http://bite-it.helsinki.fi/RUNX1.htm) | ＋ | ＋ | Homo sapiens runt-related transcription factor 1 (RUNX1) on chromosome 21(gi|224586926|ref|NG_011402.1|) | 83 | gdtca_Cluster6021 |
| [Runx2](http://bite-it.helsinki.fi/CBFA1.htm) | ＋ | － |  |  |  |
| [Runx3](http://bite-it.helsinki.fi/RUNX3.htm) | ＋ | － |  |  |  |
| [Snai1](http://bite-it.helsinki.fi/SNA.htm) | ＋ | ＋ | Bos taurus snail homolog 1 (Drosophila) (SNAI1), mRNA(gi|162951886|ref|NM_001112708.1|) | 94 | gdtca_Cluster2855 |
| [Sox9](http://bite-it.helsinki.fi/SOX9.htm) | ＋ | ＋ | Equus caballus transcription factor SOX-9 (LOC100033908), mRNA(gi|194216715|ref|XM_001498424.2|) | 96 | gdtca_Cluster3454 |
| [Sp4](http://bite-it.helsinki.fi/SP4.htm) | ＋ | － |  |  |  |
| [Sp6](http://bite-it.helsinki.fi/SP6.htm) | ＋ | － |  |  |  |
| [Tbx1](http://bite-it.helsinki.fi/TBX1.htm) | ＋ | － |  |  |  |
| [Tcf1](http://bite-it.helsinki.fi/TCF1.htm) | ＋ | － |  |  |  |
| [Tfap2a](http://bite-it.helsinki.fi/AP2A.htm) | ＋ | ＋ | Homo sapiens AP2 associated kinase 1 (AAK1), mRNA(gi|148277036|ref|NM_014911.3|) | 93 | gdtca_Cluster2807 |
| [Tfap2c](http://bite-it.helsinki.fi/AP-22.htm) | ＋ | － |  |  |  |
| [Tlx1](http://bite-it.helsinki.fi/TLX1.htm) | ＋ | － |  |  |  |
| Wt1 | ＋ | － |  |  |  |

M（mouse） P（pig） id （identity）
