# Supplementary material for: Construction of a cDNA library for miniature pig mandibular deciduous molars
Source: BMC Dev Biol. 2014 Apr 21;14:16. doi: 10.1186/1471-213X-14-16 (PMC4021421; doi:10.1186/1471-213X-14-16)
Supplement: Additional file 8 — Known growth factor expression in mice searched in the cDNA library during tooth development. [file 1471-213X-14-16-S8.doc]

| Additional file 8. Known growth factor expression in mice searched in the cDNA library during tooth development | M | P | annotation | id | unigene |
| --- | --- | --- | --- | --- | --- |
| [activin beta A](http://bite-it.helsinki.fi/ACTBA.htm) | ＋ | － |  |  |  |
| [Bmp2](http://bite-it.helsinki.fi/BMP-2.htm) | ＋ | ＋ | Macaca mulatta BMP-2 inducible kinase (BMP2K), mRNA（gi|109074373|ref|XR_011078.1|） | 88 | gdtca_Cluster9256 |
| [Bmp3](http://bite-it.helsinki.fi/BMP-3.htm) | ＋ | － |  |  |  |
| [Bmp4](http://bite-it.helsinki.fi/BMP-4.htm) | ＋ | － |  |  |  |
| [Bmp5](http://bite-it.helsinki.fi/BMP-5.htm) | ＋ | － |  |  |  |
| [Bmp6](http://bite-it.helsinki.fi/BMP6.htm) | ＋ | ＋ | Human DNA sequence from clone RP1-119C5 on chromosome 6 Contains the 5' end of the BMP6 gene for bone morphogenetic protein 6 and a CpG island, complete sequence（gi|12330708|emb|AL135778.9|） | 85 | gdtca_Cluster8553 |
| [Bmp7](http://bite-it.helsinki.fi/BMP-7.htm) | ＋ | － |  |  |  |
| [Eda](http://bite-it.helsinki.fi/TA.htm) | ＋ | － |  |  |  |
| [Egf](http://bite-it.helsinki.fi/EGF.htm) | ＋ | － |  |  |  |
| [Fgf1](http://bite-it.helsinki.fi/FGF1.htm) | ＋ | － |  |  |  |
| [Fgf10](http://bite-it.helsinki.fi/FGF10.htm) | ＋ | ＋ | Mus musculus fibroblast growth factor 10 (Fgf10), mRNA（gi|226823275|ref|NM_008002.4|） | 99 | gdtca_Cluster6182 |
| [Fgf2](http://bite-it.helsinki.fi/FGF2.htm) | ＋ | － |  |  |  |
| [Fgf3](http://bite-it.helsinki.fi/FGF3.htm) | ＋ | ＋ | Macaca mulatta similar to Fibroblast growth factor 11 (FGF-11) (Fibroblast growth factor homologous factor 3) (FHF-3) (FGF11), mRNA（gi|109113101|ref|XM_001108934.1|） | 89 | gdtca_Cluster743 |
| [Fgf4](http://bite-it.helsinki.fi/FGF4.htm) | ＋ | － |  |  |  |
| [Fgf8](http://bite-it.helsinki.fi/FGF8.htm) | ＋ | － |  |  |  |
| [Fgf9](http://bite-it.helsinki.fi/FGF9.htm) | ＋ | － |  |  |  |
| [follistatin](http://bite-it.helsinki.fi/FOLLIP.htm) | ＋ | ＋ | Porcine follistatin gene, complete cds（gi|164461|gb|M36512.1|PIGFSB） | 96 | gdtca_Cluster8420 |
| [Gdnf](http://bite-it.helsinki.fi/GDNFII.htm) | ＋ |  |  |  |  |
| [Hgf](http://bite-it.helsinki.fi/HGF-SF.htm) | ＋ | － |  |  |  |
| [Igf1](http://bite-it.helsinki.fi/IGF1.htm) | ＋ | ＋ | Homo sapiens insulin-like growth factor 2 mRNA binding protein 2 (IGF2BP2) on chromosome 3（gi|225007548|ref|NG_011602.1|） | 85 | gdtca_Cluster1638 |
| [midkine](http://bite-it.helsinki.fi/MKRNA.htm) | ＋ | － |  |  |  |
| [pleiotrophin](http://bite-it.helsinki.fi/HBGAM.htm) | ＋ | － |  |  |  |
| [Pthlh](http://bite-it.helsinki.fi/PTHRP.htm) | ＋ | ＋ | Sus scrofa parathyroid hormone-like hormone (PTHLH), mRNA （gi|47522655|ref|NM_213916.1|） | 98 | gdtca_Cluster13208.seq.Contig1 |
| [Sonic hedgehog](http://bite-it.helsinki.fi/SHH.htm) | ＋ | ＋ | Homo sapiens sonic hedgehog homolog (Drosophila) (SHH) on chromosome 7（gi|172044658|ref|NG_007504.1） | 98 | gdtca_Cluster7111 |
| [Tgfb1](http://bite-it.helsinki.fi/TGFB1R.htm) | ＋ | － |  |  |  |
| [Tgfb2](http://bite-it.helsinki.fi/TGFB2R.htm) | ＋ | ＋ | Homo sapiens transforming growth factor, beta 2 (TGFB2) gene, complete cds（gi|37953286|gb|AY438979.1） | 82 | gdtca_Cluster2986 |
| [Tgfb3](http://bite-it.helsinki.fi/TGFB3.htm) | ＋ | － |  |  |  |
| [Wnt10a](http://bite-it.helsinki.fi/WNT10A.htm) | ＋ | － |  |  |  |
| [Wnt10b](http://bite-it.helsinki.fi/WNT10B.htm) | ＋ | － |  |  |  |
| [Wnt3](http://bite-it.helsinki.fi/WNT3.htm) | ＋ | － |  |  |  |
| [Wnt4](http://bite-it.helsinki.fi/WNT4.htm) | ＋ | － |  |  |  |
| [Wnt5a](http://bite-it.helsinki.fi/WNT-5A.htm) | ＋ | － |  |  |  |
| [Wnt6](http://bite-it.helsinki.fi/WNT6.htm) | ＋ | － |  |  |  |
| [Wnt7b](http://bite-it.helsinki.fi/WNT7B.htm) | ＋ | － |  |  |  |

M（mouse） P（pig） id （identity）
