# Supplementary material for: Construction of a cDNA library for miniature pig mandibular deciduous molars
Source: BMC Dev Biol. 2014 Apr 21;14:16. doi: 10.1186/1471-213X-14-16 (PMC4021421; doi:10.1186/1471-213X-14-16)
Supplement: Additional file 9 — Known related receptor expression in mice searched in the cDNA library during tooth development. [file 1471-213X-14-16-S9.doc]

| Additional file 9. Known related receptors expression in mice searched in the cDNA library during tooth development | M | P | annotation | id | unigene |
| --- | --- | --- | --- | --- | --- |
| [Ahr](http://bite-it.helsinki.fi/AHRR.htm) | ＋ | ＋ | Sus scrofa aryl hydrocarbon receptor nuclear translocator-like (ARNTL), mRNA（gi|147898768|ref|NM_001097425.1|） | 98 | gdtca_Cluster11260.seq.Contig1 |
| [Bono1](http://bite-it.helsinki.fi/BONO1.htm) | ＋ | － |  |  |  |
| [Edar](http://bite-it.helsinki.fi/DWNLES.htm) | ＋ | － |  |  |  |
| [EGFR](http://bite-it.helsinki.fi/EGF-R.htm) | ＋ | ＋ | Sus scrofa epidermal growth factor receptor (EGFR), mRNA（gi|47522839|ref|NM_214007.1|） | 99 | gdtca_Cluster9561 |
| [EphA7](http://bite-it.helsinki.fi/EBK.htm) | ＋ | ＋ | Bos taurus similar to Ephrin type-A receptor 7 precursor（gi|119901378|ref|XM_611161.3|） | 97 | gdtca_Cluster5945 |
| [Fgfr1](http://bite-it.helsinki.fi/FGFRA.htm) | ＋ | ＋ | Sus scrofa similar to FGFR1 oncogene partner 2 , mRNA（gi|194037780|ref|XM_001924597.1|） | 100 | gdtca_Cluster224 |
| [Fgfr2](http://bite-it.helsinki.fi/FR23B.htm) | ＋ | ＋ | Homo sapiens fibroblast growth factor receptor 2 (FGFR2) on chromosome 10（gi|254553324|ref|NG_012449.1|） | 88 | gdtca_Cluster6685 |
| [Fgfr3](http://bite-it.helsinki.fi/FR33B.htm) | ＋ | － |  |  |  |
| [Fgfr4](http://bite-it.helsinki.fi/FR4.htm) | ＋ | － |  |  |  |
| [Gfr alpha-1](http://bite-it.helsinki.fi/GFRA1.htm) | ＋ | － |  |  |  |
| [Gfr alpha-2](http://bite-it.helsinki.fi/GFRA2.htm) | ＋ | － |  |  |  |
| [integrin alpha 4](http://bite-it.helsinki.fi/INTA4.htm) | ＋ | － |  |  |  |
| [integrin alpha 6](http://bite-it.helsinki.fi/INTA6P.htm) | ＋ | ＋ | Bos taurus integrin, alpha 6 (ITGA6), mRNA（gi|158341671|ref|NM_001109981.1|） | 91 | gdtca_Cluster2420 |
| [integrin alpha v](http://bite-it.helsinki.fi/INTEGAVR.htm) | ＋ | ＋ | Homo sapiens integrin, alpha V , mRNA（gi|223468596|ref|NM_001145000.1|） | 84 | gdtca_Cluster4112 |
| [integrin beta 1](http://bite-it.helsinki.fi/INTB1.htm) | ＋ | － |  |  |  |
| [integrin beta 4](http://bite-it.helsinki.fi/INTB4R.htm) | ＋ | － |  |  |  |
| [integrin beta 5](http://bite-it.helsinki.fi/INTB5.htm) | ＋ | － |  |  |  |
| [Met proto-oncogene](http://bite-it.helsinki.fi/C-MET.htm) | ＋ | － |  |  |  |
| [MFrzb1](http://bite-it.helsinki.fi/MFRZB1.htm) | ＋ | － |  |  |  |
| [MFz6](http://bite-it.helsinki.fi/MFZ6.htm) | ＋ | － |  |  |  |
| [Neuropilin1](http://bite-it.helsinki.fi/NEUROP1.htm) | ＋ | ＋ | Homo sapiens neuropilin 1 (NRP1) gene, complete cds（gi|166706800|gb|EU332859.1|） | 87 | gdtca_Cluster11672.seq.Contig1 |
| [Neuropilin2](http://bite-it.helsinki.fi/NEUROP2.htm) | ＋ | ＋ | Homo sapiens neuropilin 2 (NRP2) gene, complete cds, alternatively spliced（gi|11934947|gb|AF281074.1|AF281074） | 92 | gdtca_Cluster13309.seq.Contig1 |
| [Notch1](http://bite-it.helsinki.fi/NOTCH1.htm) | ＋ | ＋ | Canis familiaris similar to sno, strawberry notch homolog 1, transcript variant 1 (LOC477451), mRNA（gi|73994433|ref|XM_534649.2|） | 91 | gdtca_Cluster10505 |
| [Notch2](http://bite-it.helsinki.fi/NOTCH2.htm) | ＋ | ＋ | Homo sapiens Notch homolog 2 (Drosophila) (NOTCH2) on chromosome 1（gi|193290125|ref|NG_008163.1|） | 84 | gdtca_Cluster9819 |
| [Notch3](http://bite-it.helsinki.fi/NOTCH3.htm) | ＋ | － |  |  |  |
| [Patched 1](http://bite-it.helsinki.fi/PATCH.htm) | ＋ | ＋ | Homo sapiens patched homolog 1 (Drosophila) (PTCH1) on chromosome 9（gi|187940264|ref|NG_007664.1|） | 91 | gdtca_Cluster4085| |
| [Patched 2](http://bite-it.helsinki.fi/PATCHED2.htm) | ＋ | － |  |  |  |
| [Pthr1](http://bite-it.helsinki.fi/PTHRPR.htm) | ＋ | － |  |  |  |
| [Pvrl1](http://bite-it.helsinki.fi/PVRL1.htm) | ＋ | ＋ | Homo sapiens poliovirus receptor-related 1 (PVRL1) on chromosome 11（gi|261278322|ref|NG_013083.1|） | 89 | gdtca_Cluster4892 |
| [Rara](http://bite-it.helsinki.fi/RARA.htm) | ＋ | － |  |  |  |
| [Rarb](http://bite-it.helsinki.fi/RARB.htm) | ＋ | ＋ | Equus caballus retinoic acid receptor, beta, transcript variant 2 (RARB), mRNA（gi|194221542|ref|XM_001494142.2） | 93 | gdtca_Cluster9538 |
| [Rarg](http://bite-it.helsinki.fi/RARG.htm) | ＋ | － |  |  |  |
| [Ret](http://bite-it.helsinki.fi/RETMO.htm) | ＋ | ＋ | Sus scrofa similar to ret proto-oncogene (LOC100153852), mRNA（gi|194042653|ref|XM_001926369.1） | 99 | gdtca_Cluster5320 |
| [Robo2](http://bite-it.helsinki.fi/ROBO1.htm) | ＋ | ＋ | Pan troglodytes similar to Roundabout homolog 2 precursor, transcript variant 5 (ROBO2), mRNA（gi|114587934|ref|XM_001144482.1|） | 87 | gdtca_Cluster6311 |
| [Robo1](http://bite-it.helsinki.fi/ROBO2.htm) | ＋ | ＋ | Homo sapiens roundabout, axon guidance receptor, homolog 1 (Drosophila) (ROBO1) on chromosome 3（gi|225903388|ref|NG_011729.1|） | 91 | gdtca_Cluster10824 |
| [Ror1](http://bite-it.helsinki.fi/ROR1.htm) | ＋ | ＋ | Human DNA sequence on chromosome 1p31.1-31.3 Contains part of the ROR1 gene（ gi|7801530|emb|AL137859.3|） | 87 | gdtca_Cluster9953 |
| [Ror2](http://bite-it.helsinki.fi/ROR2.htm) | ＋ | ＋ | Homo sapiens discoidin domain receptor tyrosine kinase 2 (DDR2) on chromosome 1（gi|282721095|ref|NG_016290.1|） | 80 | gdtca_Cluster12576.seq.Contig1 |
| [Rxra](http://bite-it.helsinki.fi/RXRA.htm) | ＋ | － |  |  |  |
| [Rxrb](http://bite-it.helsinki.fi/RXRB.htm) | ＋ | － |  |  |  |
| [Rxrg](http://bite-it.helsinki.fi/RXRG.htm) | ＋ | － |  |  |  |
| [Tgfbr2](http://bite-it.helsinki.fi/TGFBRII.htm) | ＋ | ＋ | Sus scrofa transforming growth factor beta type 2 receptor (TGFBR2) gene, complete cds（gi|108755893|gb|DQ519377.1|） | 97 | gdtca_Cluster9487 |
| [Tnfrsf19](http://bite-it.helsinki.fi/TROY.htm) | ＋ | － |  |  |  |

M（mouse） P（pig） id （identity）
